# Comparing moderate–severe and severe mitral regurgitation in transcatheter aortic valve replacement on 1-year survival: insights from a Japanese Nationwide Registry

**DOI:** 10.1007/s00380-024-02491-6

**Published:** 2024-12-19

**Authors:** Kaoru Matsuura, Hiraku Kumamaru, Shun Kohsaka, Tomoyoshi Kanda, Hideki Kitahara, Kazuo Shimamura, Yoshio Kobayashi, Goro Matsumiya

**Affiliations:** 1 Department of Cardiovascular Surgery, Chiba University Graduate School of Medicine, Chiba University Hospital, 1-8-1 Inohana, Chuo Ward, Chiba, Chiba 260-0856 Japan; 2 Department of Healthcare Quality Assessment, Graduate School of Medicine, The University of Tokyo, Tokyo, Japan; 3 Department of Cardiology, Keio University School of Medicine, Tokyo, Japan; 4 Department of Cardiology, Chiba University Graduate School of Medicine, Chiba, Japan; 5 Department of Cardiovascular Surgery, Osaka University Graduate School of Medicine, Suita, Japan

**Keywords:** Transcatheter, Aortic valve replacement/implantation, Outcomes, Prognosis

## Abstract

This study aims to compare 1-year outcomes after transcatheter aortic valve replacement (TAVR) between patients with moderate–severe MR and severe MR preoperatively using the Japan Transcatheter Valve Therapy (J-TVT) registry. Patients undergoing TAVR for aortic stenosis between August 2013 and December 2019 with preoperative mitral regurgitation of moderate–severe (group MR3) or severe (group MR4) were included. Patients with a history of valve surgery and dialysis patients were excluded. A total of 2017 patients were included, and 1-year follow-up data were obtained from the registry (follow-up rate 98.5%). Propensity-score matching between MR3 and MR4 groups was performed. All-cause mortality and the composite outcome of death and/or heart failure events were compared. Crude data showed that 1-year survival was significantly higher in the MR 3 (89.8%) than MR 4 (84.7%) groups, and freedom from 1-year mortality and heart failure events was also higher in the MR 3 (87.1%) than MR 4 (80.5%) groups (*p* = 0.0001). After propensity-score matching, 452 cases (226 cases each in MR 3 group and MR 4 group) were extracted. Cox regression model showed no statistical difference in the 1-year survival rate between MR 3 group (84.5%) and MR 4 group (85.5%) (*p* = 0.84), nor in freedom from 1-year death and/or heart failure events between MR 3 group (80.2%) and MR 4 group (81.6%) (*p* = 0.72). The 1-year survival rate and freedom from death and/or heart failure events were found to be similar between patients undergoing TAVR with MR grade 3 and MR grade 4.

## Introduction

The utilization of transcatheter aortic valve replacement (TAVR) has become more common, facilitating therapeutic interventions for patients with severe aortic stenosis (AS). However, an increasing number of older patients also have concurrent valvular diseases, particularly mitral regurgitation (MR) [1–7]. MR in patients undergoing transcatheter aortic valve replacement (TAVR) can significantly influence their outcomes. Indeed, in a previous nationwide study utilizing the Japan Transcatheter Valve Therapy (J-TVT) database, we investigated the impact of preoperative MR that was more than mild on 1-year outcomes [8]. Specifically, our analysis compared the 1-year outcomes of patients with preoperative MR grades 0–2 (none to moderate) and grades 3–4 (moderate–severe to severe), revealing no difference in mortality but a discrepancy in the composite outcome. However, the precise impact of grade 3 vs grade 4 MR on long-term outcomes remains unknown. This raised the possibility of a divergence in prognosis between patients with truly severe MR (grade 4) and those with moderate–severe MR (grade 3). Understanding this distinction is crucial as a substantial number of patients have grade 3 MR and often present clinically challenging profiles preoperatively. This study aims to compare the 1-year prognoses of patients with preoperative moderate–severe MR (grade 3) and severe MR (grade 4).

## Materials and methods

### Data source

This study utilized data from the J-TVT Registry, which is a comprehensive, nationwide database established through collaboration between four Japanese academic societies: the Japanese Society of Cardiology, the Japanese Society of Cardiovascular Surgery, the Japanese Society of Thoracic Surgery, and the Japanese Society of Cardiovascular and Interventional Therapy. The registry, in partnership with the Pharmaceuticals and Medical Devices Agency (PMDA), encompasses clinical datasets from all institutions performing TAVR [9]. To ensure data quality, the registry mandates the registration of all TAVR cases by participating institutions and operators. Institutions are required to maintain complete case registration and undergo a renewal process every 3 years for institutional certification. These measures contribute to the robustness of the J-TVT registry data. At each participating hospital, a designated data manager is responsible for electronically registering the clinical data on the central server. The central office conducts regular monitoring to ensure comprehensive and accurate data entry. Informed consent for data registration is obtained from all patients at each participating hospital using an opt-out approach. Data collection for the J-TVT registry began in September 2013. The use of data for the present study was approved by the academic committee of J-TVT, and the study design, including the data registration project, received ethical approval from the Institutional Review Board of Chiba University Graduate School of Medicine (Ethical Committee No. 3744).

### Data collection and outcome definitions

**Fig. 1:**
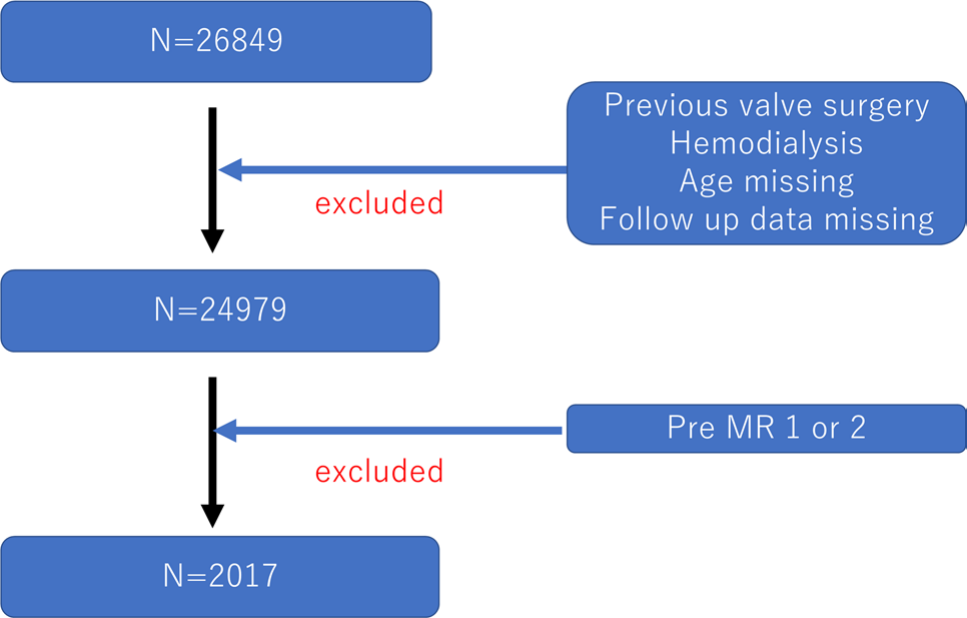
Patient selection flowchart. *MR* mitral regurgitation

This study reviewed patient-level data from a total of 26,849 first-time transcatheter aortic valve replacement (TAVR) procedures performed between August 2013 and December 2019. Patients who had previously undergone valve surgery and those on chronic hemodialysis were excluded from the analysis. In the J-TVT　registry, the severity of mitral regurgitation (MR) was classified into five grades: none or trivial (grade 0), mild (grade 1), moderate (grade 2), moderate–severe (grade 3), and severe (grade 4), based on standard definitions of mitral valve disease using echocardiography. This study specifically focused on patients with preoperative MR grade 3 and preoperative MR grade 4. The primary outcome measure was all-cause mortality, and the secondary outcome measure was a composite outcome comprising death and/or rehospitalization due to heart failure during the 1-year period following the TAVR procedure. Patient selection flowchart is described in Fig. 1.

### Statistical methods

All continuous values are expressed as mean ± standard deviation or median + inter-quartile range as appropriate. Patients were categorized into two groups based on their mitral regurgitation (MR) grade: MR grade 3 (MR 3 group) and MR grade 4 (MR4 group). A logistic regression model was used to calculate the propensity score for being in the MR4 group, taking into account various background factors including age, gender, body surface area, non-elective surgery, hypertension, dyslipidemia, diabetes mellitus, chronic lung disease grade 3–4, coronary artery disease, non-cardiac artery disease, previous percutaneous coronary artery intervention, previous coronary artery bypass grafting, previous cerebrovascular event, pacemaker implantation, aortic calcification, malignancy, hemoglobin, albumin, mean aortic valve pressure gradient, left ventricular ejection fraction, aortic regurgitation grade 3–4, bicuspid valve, and trans-femoral approach. Propensity score matching was performed at a 1:1 ratio using the nearest neighbor matching method without replacement, with a caliper of 0.2 SD of the logit propensity score. The clinical characteristics before and after propensity score matching were compared using the standardized mean difference. The Kaplan–Meier method was utilized to assess the incidence of outcomes during the 1-year post-procedure period, and the log-rank test was employed to compare the incidence between the two groups. Two-sided statistical tests were employed, and a *p* value of less than 0.05 was considered statistically significant. And Cox regression model was used to compare two groups. All statistical analyses were conducted using SAS version 9.4 (SAS Institute, Cary, NC, USA).

## Results

A total of 1,776 patients were in the MR 3 group, while 241 patients were in the MR 4 group. The baseline characteristics of the patients are summarized in Table 1. Before matching, the average age was 85.1 ± 5.3 years in the MR 3 group and 85.2 ± 5.5 years in the MR 4 group. The percentages of patients in NYHA class 3–4 were 38.4% in the MR 3 group and 52.7% in the MR 4 group. Non-elective surgery was performed in 6.0% of the MR 3 group and 11.6% of the MR 4 group. The average left ventricular ejection fraction was 55.4 ± 14.8% in the MR 3 group and 53.1 ± 17.0% in the MR 4 group. Aortic insufficiency grade 3–4 was present in 20.7% of the MR 3 group and 19.5% of the MR 4 group. The transfemoral approach was employed in 90.3% of the MR 3 group and 91.7% of the MR 4 group. The prevalence of chronic lung disease grade 3–4 was 7.4% in the MR 3 group and 12.0% in the MR 4 group.

**Table 1:** Patient characteristics (pre-matching and post-matching)

		Pre-matching					Post-matching				
		MR3		MR4			MR3		MR4		
N		1776		241		SMD	226		226		SMD
Age	Mean (SD)	85.1	5.3	85.2	5.5	0.01384	84.8	5.4	852	5.5	0.079
Gender	Male (%)	521	29.3%	58	24.1%	− 0.11932	55	24.3%	56	24.8%	0.010
BSA	Mean (SD)	1.41	0.16	1.37	0.17	− 0.2132	1.37	0.17	1.37	0.18	− 0.010
NYHA 3–4	%	682	38.4%	127	52.7%	0.29007	122	54.0%	116	51.3%	− 0.053
Non elective	%	106	6.0%	28	11.6%	− 0.2005	19	8.4%	22	9.7%	− 0.046
Comorbidities	Hypertension	1346	75.8%	183	75.9%	0.0034	164	72.6%	172	76.1%	0.081
(%)	Hyperlipidemia	812	45.7%	102	42.3%	− 0.06847	91	40.3%	93	41.2%	0.018
	DM	425	23.9%	64	26.6%	0.06047	46	20.4%	58	25.7%	0.126
	Chronic lung disease grade 3–4	132	7.4%	29	12.0%	0.15569	27	11.9%	26	11.5%	− 0.014
	CAD	583	32.8%	77	32.0%	− 0.01873	71	31.4%	74	32.7%	0.028
	Non cardiac artery disease	220	12.4%	33	13.7%	0.03878	36	15.9%	31	13.7%	− 0.062
	PCI history	412	23.2%	57	23.7%	0.0107	50	22.1%	55	24.3%	0.052
	CABG history	106	6.0%	17	7.1%	0.04401	14	6.2%	17	7.5%	0.053
	Past cerebrovascular accident	130	7.3%	36	14.9%	0.24403	30	13.3%	31	13.7%	0.013
	Pacemaker	132	7.4%	20	8.3%	0.03219	25	11.1%	20	8.8%	− 0.074
	Aortic calcification	180	10.1%	18	7.5%	− 0.09421	18	8.0%	18	8.0%	0.000
	Malignancy	140	7.9%	22	9.1%	0.04467	25	11.1%	20	8.8%	− 0.074
Lab data	Hb, mean (SD)	11.1	1.8	10.6	1.5	− 0.30595	10.5	1.4	10.6	1.5	0.058
	Alb, mean (SD)	3.7	2.3	3.4	0.5	− 0.20381	3.4	0.6	3.4	0.5	0.111
	Cre, mean (SD)	1.13	1.08	1.24	0.81	0.11692	1.2	0.8	1.2	0.8	0.037
TTE data	Mean gradient, mean (SD)	49.2	20.3	46.2	20.4	− 0.14898	47.5	18.4	46.2	20.2	− 0.072
	LVEF,mean (SD)	55.4	14.8	53.1	17	− 0.14309	53.2	15.7	53.3	16.7	0.005
	Aortic insufficiency 3–4(%)	368	20.7%	47	19.5%	− 0.03041	49	21.7%	45	19.9%	− 0.044
	Bi-cuspid valve (%)	56	3.2%	6	2.5%	− 0.04008	6	2.7%	6	2.7%	0.000
Approach	Transfemoral (%)	1603	90.3%	221	91.7%	0.05036	206	91.2%	207	91.6%	0.016

*MR* mitral regurgitation, *BSA* body surface area, *NYHA* New York Heart Association, *DM* diabetes mellitus, *CAD* coronary artery disease, *PCI* percutaneous catheter intervention, *CABG* coronary artery bypass graft, *Hb* hemoglobin, *Alb* albumin, *Cre* creatine, *LVEF* left ventricular ejection fraction, *TTE* transthoracic echocardiography, *SD* standard deviation, *SMD* standardized mean difference

**Fig. 2:**
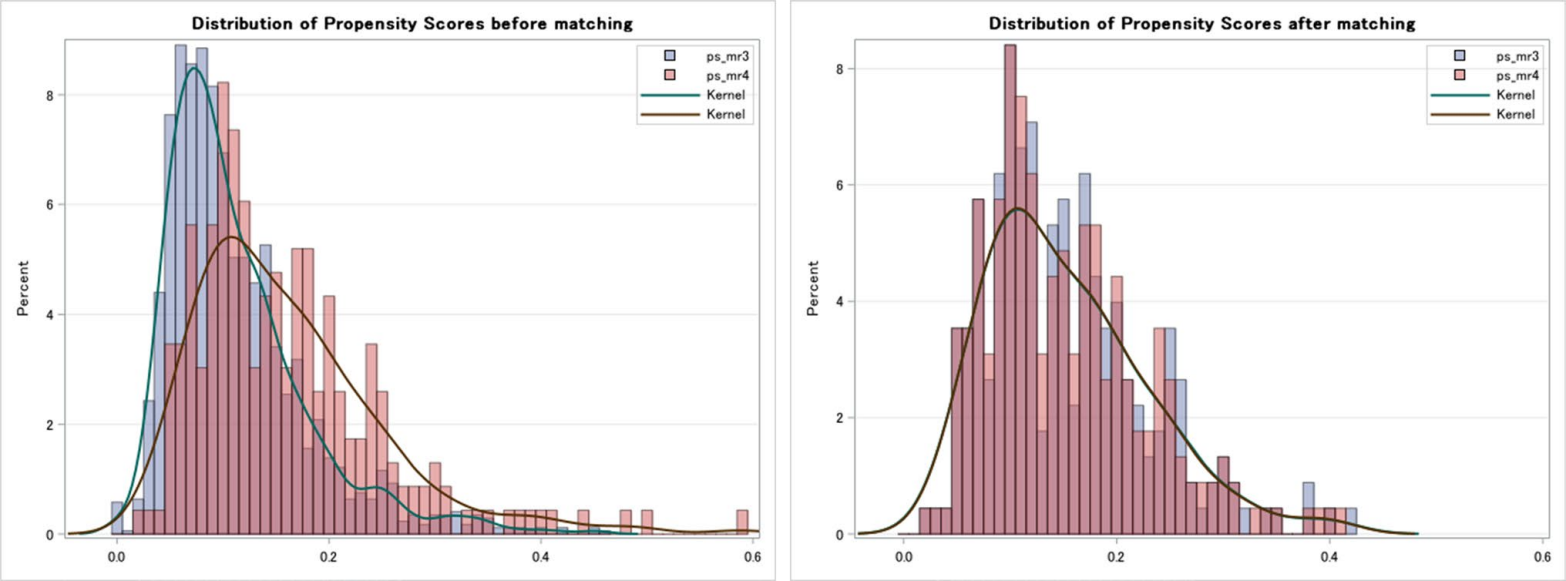
The distribution of the propensity score before and after matching. *MR* mitral regurgitation

After propensity score matching, a total of 452 cases (226 in each group) were matched. The C statistics of the propensity score was 0.685. The distribution of the propensity score matching before and after matching is shown in Fig. 2. The average age was 84.8 years in the MR 3 group and 85.2 years in the MR 4 group. The percentage of males was 24.3% in the MR 3 group and 24.8% in the MR 4 group. The preoperative NYHA class 3–4 was 54.0% in the MR 3 group and 51.3% in the MR 4 group. The transfemoral approach was employed in 91.2% of the MR 3 group and 91.6% of the MR 4 group.

**Fig. 3:**
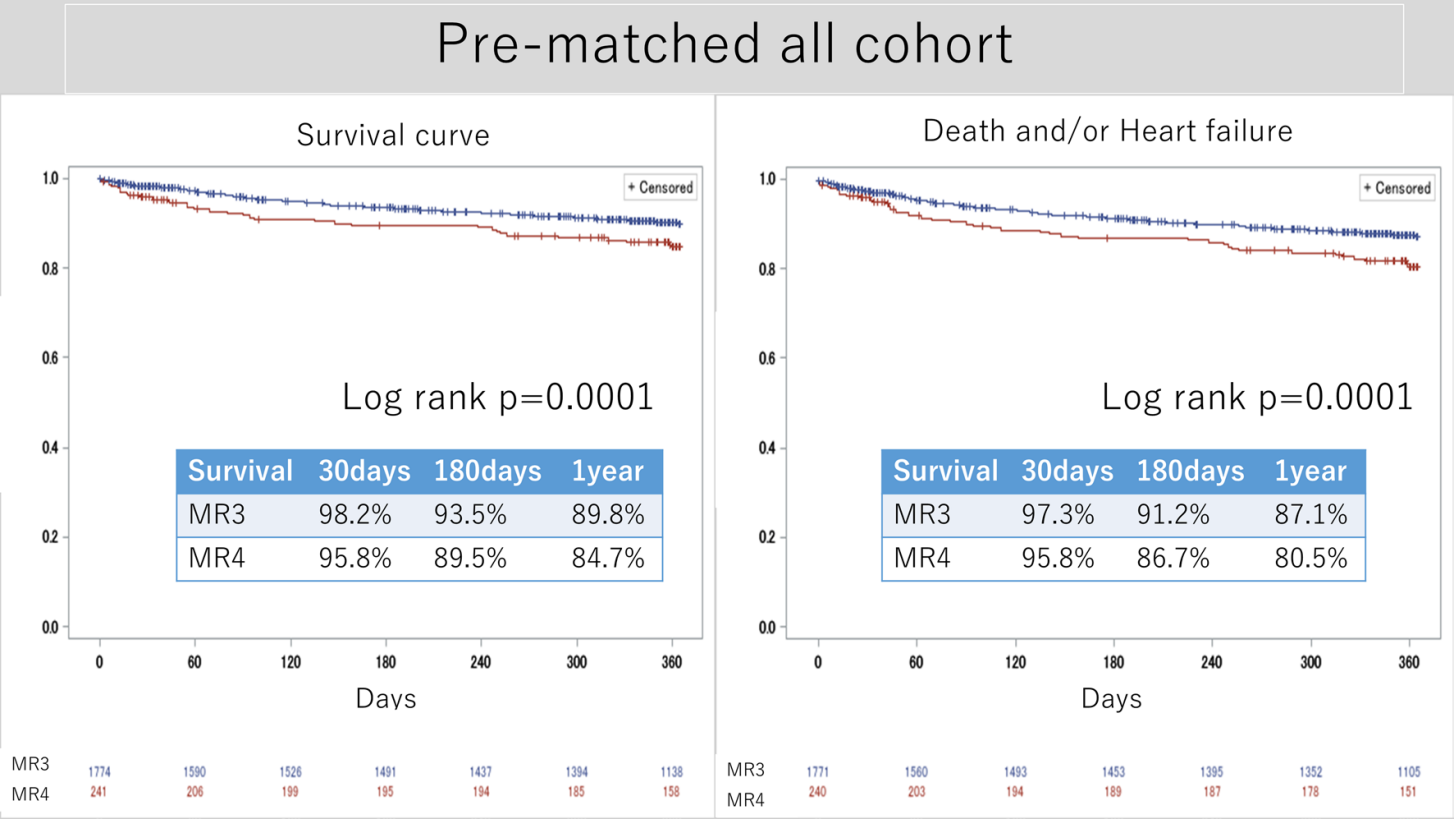
Survival curve and survival + heart failure (pre-matched cohort). *MR* mitral regurgitation

**Fig. 4:**
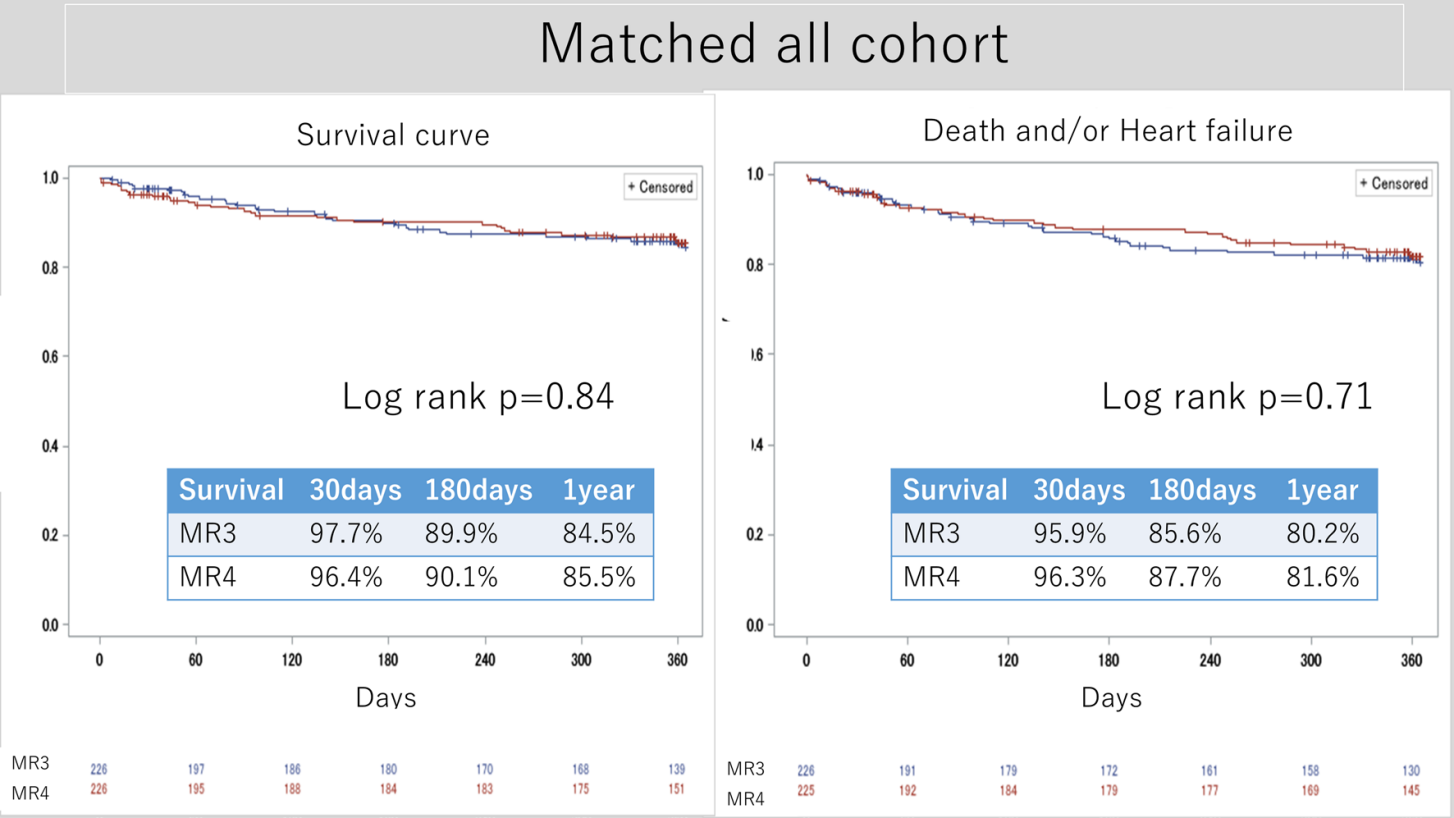
Survival curve and survival + heart failure (all matched cohort). *MR* mitral regurgitation

Figure 3 shows the Kaplan–Meier curve of survival and composite outcomes in the pre-matched cohort. The 1-year survival rate was 89.8% in the MR 3 group and 84.7% in the MR 4 group (*p* = 0.0001), while the freedom from composite outcome was 87.1% in the MR 3 group and 80.5% in the MR 4 group (*p* = 0.0001). Figure 4 presents the Kaplan–Meier curve of survival and freedom from the composite outcome in all matched cohorts. The 1-year survival rate was 84.5% in the MR 3 group and 85.5% in the MR 4 group, with no significant difference between the matched groups (*p* = 0.84). The 1-year freedom from the composite outcome was not different between the MR 3 group (80.2%) and the MR 4 group (81.6%) (*p* = 0.71).

## Discussion

The main findings of this study highlight the differences in outcomes between patients with moderate–severe MR and severe MR undergoing TAVR. Patients with severe MR had lower 1-year survival rates and a higher composite outcome, including heart failure hospitalization. However, after propensity score matching, there was no significant difference in survival or composite outcome between the two groups. In other words, after adjustment for confounders, comparable 1-year outcomes can be expected between patients with grade 3 and 4 MR after TAVR. On the other hand, data for comparison with conservative treatment were not obtained in this study and is a subject for future research. One possible reason for the lack of difference in prognosis after PS matching between the patients with moderate–severe MR and severe MR could be that the degree of improvement in cardiac function and MR is comparable between them. There may also, of course, be a problem with the analysis of PS matching itself.

The decision-making process for patients with coexisting MR and aortic stenosis (AS) requires careful evaluation and clinical judgment. Severe MR often necessitates double valve surgery, but in certain cases, minimally invasive TAVR alone may be appropriate. This study provides valuable clinical data for physicians performing TAVR and helps guide treatment decisions based on the severity of MR.

The comparison between severe MR and moderate–severe MR in this study holds significant clinical implications. In practice, there appears to be a notable difference in preoperative decision-making for patients with severe MR versus those with moderate–severe MR. When a patient has severe MR, double valve surgery should be strongly considered as the preferred treatment option. However, depending on the patient's condition, there may be instances where minimally invasive TAVR alone is the preferred choice [10]. Conversely, for patients with moderate–severe MR, TAVR alone may be a reasonable option to improve their condition, particularly if there are certain surgical risks involved. This study aimed to determine whether there is a distinct difference between moderate–severe MR and severe MR using real-world clinical data, and we believe that this information provides valuable insights for physicians performing TAVR procedures.　On the other hand, it is worth noting that MR can sometimes worsen significantly during TAVR due to various mechanisms, and this consideration should be kept in mind during the procedure [11].

The prevalence of MR in the Japanese TAVR registry was 8.0%, with 11.9% classified as severe MR and 88.1% as moderate–severe MR. This is comparable to findings from the US TAVR Registry, although variations in patient populations and selection criteria may account for the differences observed [12]. Other studies have also reported the prevalence of MR in the setting of TAVR, most have reported that about 10% of all TAVI cases are complicated by moderate–severe MR or higher [5, 13]. Based on the pre-matching data in this study, patients with severe MR exhibited more severe symptoms compared to patients with moderate–severe MR. For instance, 40% of patients with severe MR had moderate–severe tricuspid regurgitation (TR), 12% had grades 3–4 chronic pulmonary disease, and 40% had pulmonary hypertension, indicating a significant degree of comorbidity and poor preoperative condition in patients with severe MR. In this sense, TAVR alone seems appropriate for certain patients with severe MR.

Regarding the speculation regarding the differences in 1-year survival and heart failure hospitalizations before and after matching, one possible explanation for this outcome is that matching for preoperative condition attenuated the impact of severe MR, thereby eliminating the observed differences. Nevertheless, considering the substantial comorbidities and poor preoperative condition often seen in patients with severe MR, the results obtained from the crude data remain clinically important.

Previously, Miura et al., utilizing the Japanese OCEAN-TAVR registry, demonstrated that moderate or severe MR was associated with an increased risk of all-cause mortality at the 2-year mark following TAVR [14]. There are several differences between their study and ours. First, our study utilized a national database encompassing all TAVR cases in Japan, whereas the OCEAN-TAVR registry included only cases performed at 14 specific centers. Second, our study included a substantially larger number of cases (24,979 cases) compared to the OCEAN-TAVR registry's 1587 cases. Additionally, the previous study did not conduct a sub-analysis differentiating between moderate–severe MR and severe MR. Therefore, we believe that the results obtained from our study provide valuable and significant information for clinical practice in real-world settings.

Although the JTVT database study did not differentiate between functional and degenerative MR, it is evident that the etiology of MR may influence the response to TAVR. Kiramaijyan et al. reported similar short- and long-term survival outcomes between patients undergoing TAVR with functional or organic MR [13]. However, other previous reports have indicated that patients with moderate or severe degenerative MR have an increased risk of long-term mortality [15]. Kato et al. found that TAVR-mediated improvement of degenerative MR contributed to a better prognosis, whereas improvement in functional MR was not associated with prognosis [16]. Doldi et al. separately investigated functional MR in terms of atrial and ventricular functional MR [17]. They reported MR improvement rates of 80% for atrial functional MR, 69% for ventricular functional MR, and 40% for degenerative MR [17]. The extent of degenerative MR inclusion in the study cohort and the degree of improvement in functional MR remain unknown. Further research is warranted to explore these aspects.

Currently, due to the lack of randomized clinical trials, evidence-based recommendations on intervention for MR complications cannot be made. Feldt et al. reported that mild improvement in MR with TAVR is associated with lower long-term mortality, whereas worsening MR is linked to a two-fold increase in mortality [15]. Several studies have investigated the prognosis of moderate to severe MR left untreated during surgical aortic valve replacement and found that both early and late outcomes are worse [18, 19]. However, the clinical consequences of leaving MR untreated during TAVR are still inconsistent. Some recent meta-analyses have indicated that patients with significant MR experience higher mortality rates at 30 days, 1 year, and 2 years after TAVR, while others have not observed such associations [7, 10, 20]. Nevertheless, the present study, based on a nationwide registry and rigorous statistical matching, provides valuable insights into the decision-making process for performing TAVR in patients with severe MR in the modern era. Additionally, conducting a thorough evaluation of the underlying cause of complicating MR before TAVR and optimizing medical therapy, regardless of the etiology, would be beneficial.

Further with the rapid development of less invasive transcatheter treatments for MR, there is a potential for simultaneous treatment of multiple valves using transcatheter technology, which may become a viable option in the future [21]. As techniques like transcatheter edge-to-edge repair and transcatheter mitral valve replacement advance, more patients may be eligible for treatment with transcatheter aortic valve implantation (TAVI) alone, and their MR can be monitored to assess improvement.

## Limitations

This study had several limitations that should be acknowledged. First, the study was unable to differentiate between functional and degenerative MR, which could have implications for the response to TAVR because the J-TVT registry does not collect information on MR etiology. The inconsistency component of MR assessment is one of limitations as MR assessment varies from facility to facility and from timing to timing of the examination. Second, the clinical outcome data was limited to a 1-year follow-up period due to the available data in the J-TVT registry at the time of the study. Additionally, follow-up echocardiographic data, including information on residual MR, were not available in the registry. Considering that the improvement of MR with TAVR may have an impact on prognosis, further research is necessary to investigate this relationship. These limitations highlight the need for future studies to provide a more comprehensive understanding of the association between MR and TAVR outcomes.

## Conclusions

This study found that 1-year survival and heart failure rates after TAVR were similar for patients with concomitant moderate–severe MR and those with severe MR.

## Data Availability

This is an national database study. So data itself is not availavle for readers.
